# Halogenated Metabolites from the Diet of *Aplysia dactylomela* Rang

**DOI:** 10.3390/molecules25040815

**Published:** 2020-02-13

**Authors:** Kishneth Palaniveloo, Mohammed Rizman-Idid, Thilahgavani Nagappan, Shariza Abdul Razak

**Affiliations:** 1 Institute of Ocean and Earth Sciences, Institute for Advanced Studies Building, University of Malaya, Wilayah Persekutuan Kuala Lumpur 50603, Malaysia; rizman@um.edu.my; 2 School of Marine and Environmental Sciences, Universiti Malaysia Terengganu, Kuala Terengganu 21030, Terengganu, Malaysia; thila.vani@umt.edu.my; 3 Institute of Marine Biotechnology, Universiti Malaysia Terengganu, Kuala Terengganu 21030, Terengganu, Malaysia; 4 Nutrition and Dietetics Program, School of Health Sciences, Health Campus, Universiti Sains Malaysia, Kubang Kerian 16150, Kelantan, Malaysia

**Keywords:** sea hare, invertebrate, halogenated, secondary metabolite, bio-activity

## Abstract

Invertebrates are an important source of structurally-diverse and biologically-active halogenated metabolites. The sea hare *Aplysia dactylomela* Rang has long been known to possess halogenated metabolites of dietary origin that are used as a self-defense mechanism. The compounds from *Aplysia dactylomela* Rang are comprised mainly of terpenoids and small percentages of C-15 acetogenins, indoles, macrolides, sterols and alkaloids with potent cytotoxic, anti-microbial and anti-inflammatory properties. For decades the metabolites discovered have been investigated for their medical and pharmaceutical applications, so much so that the ecological role of the metabolites has been overlooked. The interaction between *Aplysia dactylomela* Rang and its diet that is comprised of seaweed can provide information into the distribution and diversity of the seaweed, the application of bioaccumulated secondary metabolites as part of its defense mechanism and the potential roles of these metabolites for adaptation in the marine environment. This paper compiles the diversity of halogenated secondary metabolites documented from *Aplysia dactylomela* Rang.

## 1. Sea Hare *Aplysia dactylomela* Rang

Sea hares are gastropod mollusks that supply one of the richest sources of halogenated secondary metabolites among marine invertebrates [1]. Since the 1960s sea hares have been investigated for their diet-derived halogenated secondary metabolites. With greatly reduced shell and potential vulnerability to predators, these organisms are forced to display effective chemical defense. These chemicals are sequestered from their diet [2] and stored in tissues exposed to predation [3].

*Aplysia dactylomela* Rang is known to selectively feed on the red algae genus *Laurencia* that produces a diverse range of halogenated secondary metabolites [4]. It is also known to bio-accumulate and sequester metabolites through transformation of existing compounds for defenses against predators [5]. The relationship between the sea hare and red algae is also shown in the purplish chemical ink it secretes as part of its defense strategy. The ink consists of the chemicals opaline, aplysioviolin and phycoerythrobilin, which is derived from a light harvesting protein commonly found in the red algae [6]. Opaline functions as an anti-predatory tool by interfering the olfactory and non-olfactory chemical sense of predators, while the purplish color of the chemical ink is obtained from the compounds aplysioviolin and phycoerythrobilin [7]. The seaweed derived metabolites, mostly halogenated, are of ecological importance to the sea hares as a tool defense against pathogens and fouling organisms [4]. Such metabolites are also of great ecological importance to other tropical benthic organisms in coral reefs due to high rate of attacks by predators [8].

A large variety of halogenated secondary metabolites have been isolated from both temperate and tropical sea hares [9,10,11]. Halogenated metabolites from sea hares are categorized into terpenes, alkaloids and poly-phenols [8], and are made up of eudesmane, chamigrane, cuparane, dollabellane diterpene, C-15 ethereal lipid [12], C-15 acetogenin [13] and a syndrean [10,14] type skeleton. The secondary metabolites from *Aplysia dactylomela* Rang have also been reported to display potent cytotoxic, antimicrobial, antifungal, neurotoxic and antibiotic compounds [2,15], contributing to the development of pharmaceutical drugs. Statistics reveal that approximately 56% of new active marine natural products exhibit anti-cancer properties, followed by many which are anti-bacterial (13%), and as many as 18 mollusk-derived metabolites are already in the pipeline of drug development [16]. From an ecological perspective, the diet preference of the sea hare provides an interesting insight into the interaction between sea hares and seaweed. This in return has led to the start of studies to observe the biochemical changes in sessile benthic organisms such as the nudribranchs, sea urchin, sea cucumber and microbes through the modification of diet patterns. Plenty of reviews on marine natural compounds are currently available [16] with no particular emphasis on halogenated metabolites of the sea hare. This review compiles literature on *Aplysia dactylomela* Rang diet-derived halogenated compounds since the 1960s along with some of their notable bio-activities.

## 2. The Role of Halogenated Metabolites for *Aplysia dactylomela* Rang

The survival of marine organisms in the marine environment is dependent on the strategies these organisms develop to adapt to and live in extreme environments. Due to competition for space, light and nutrients, most sessile and slow-moving organisms have naturally evolved to produce a complex array of biologically active metabolites. The sea hare *Aplysia dactylomela* Rang develops its defense strategy by bio-accumulating halogenated secondary metabolites from its diet [17]. The accumulation of diet-derived metabolites in the mantle and mucus of sea hares are important for chemical defense. Metabolites are concentrated along the mantle border, in mantle dermal formations, by several species of sea hares, often in their natural forms without being transformed. This strategy was developed by sea hares with the aims of: (1) maximizing the defensive effects by concentrating the chemicals; (2) avoiding auto-toxicity; (3) concentrating the compounds near the surface to facilitate excretion into mucus [18]. However recently, secondary metabolites have been linked to marine biodiversity on genetic, species and ecosystem levels [19]. A halogenated secondary metabolite can act as an important taxonomic tool at the species level [20]. For years, interest in natural products has been focused on meeting biomedical applications for new drugs against fungal, parasitic, bacterial and viral diseases [21]. Interestingly, these molecules were found to control interactions between organisms, influencing population structure and ecosystem function [22]. Currently, secondary metabolites are used to assess ecological aspects such as chemical sensing of the environment, intra-specific signaling, allelopathy, predator-prey and host-parasite interactions, bio-accumulation and transfer of toxins within food web. Diet derived metabolites can be a measure of the availability of a species or a source of food in a reef [17]. Interest in herbivory has highlighted interactions between algae and herbivorous invertebrates. The increased trend toward this research niche shows the need for these ecological interactions to be studied to understand the ecological role of secondary metabolites in the host organism.

## 3. Biogenesis of Halogenated Metabolites

Terpenes consist of isoprene units. Two isoprene units form the basic monoterpene with the molecular formula C_10_H_15_. Sesquiterpenes consists of three isoprene units, with the primary molecular formula C_15_H_24_. A large percentage of marine-algal-derived secondary metabolites are categorized under this class. A sesquiterpene sub-class, C-15 acetogenins, contain a unique group of metabolites that is only present among marine algae and some invertebrates. With each additional isoprene unit, the metabolites are group accordingly. A four isoprene unit metabolite is classified as diterpene (C_20_H_32_), followed by sesterterpene (C_25_H_40_) and triterpene (30 carbons) [23,24].

**Figure 1:**
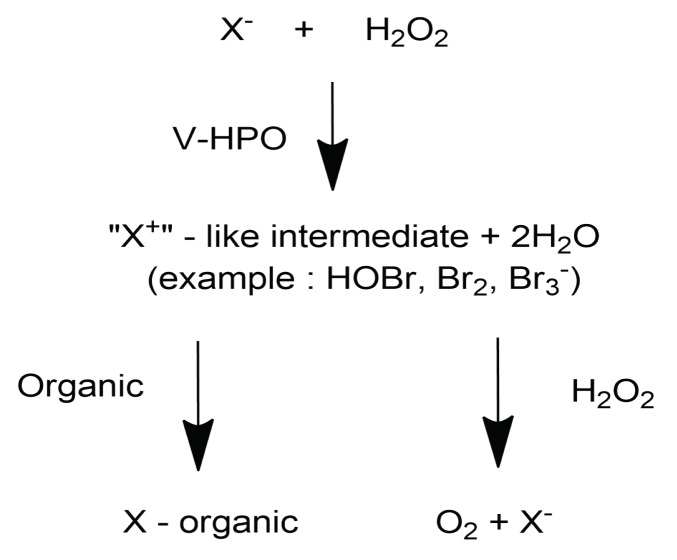
Proposed reaction scheme of vanadium haloperoxidase.

The biogenesis of halogenated terpenes is modulated by haloperoxidase enzymes. Haloperoxidases have been reported from a variety of marine organisms, and vanadium-dependent haloperoxidases (V-HPOs) are the most prevalent. V-HPOs have now isolated and purified from marine algae that produce halogenated secondary metabolites [25]. V-HPOs catalyze the oxidation of halides (iodide, bromide, chloride) by hydrogen peroxide. Hydrogen peroxide (H_2_O_2_) is present in the marine environment as a reactive intermediate [26]. V-HPOs are classified according to the electronegativity of the halogen they oxide. As such, vanadium chloroperoxidase (V-ClPO) is able to oxidize chloride, bromide and iodide, while vanadium bromoperoxidase (V-BrPO) is associated with bromide and iodide. V-BrPO is an enzyme present in marine algae that catalyzes electrophilic halogenation reactions using hydrogen peroxide to oxidize and activate bromide ion. Currently, only V-BrPO has been isolated and characterized from all classes of marine algae, while V-ClPO was reported from a dematiaceous hyphomycete fungi [27]. The oxidation of halides involves a two-step process where in the first step, V-HPOs catalyze the oxidation of halides by hydrogen peroxide producing a two electron oxidized halogen intermediate. The second step resumes with the oxidized intermediate halogenating an appropriate organic substrate or reacting with another equivalent of hydrogen peroxide, forming dioxygen in the singlet-excited state. Plenty of X-ray structure investigations are being done: haloperoxidases are being isolated and cloned to study the reactivity of haloperoxidases and their mechanism of action. Understanding is still basic and further research is needed to deeply understand the biogenesis of halogenated metabolites. A summary of proposed reaction scheme of vanadium haloperoxidase is shown in Figure 1.

## 4. Halogenated Secondary Metabolites of *Aplysia dactylomela* Rang

The sea hare *Aplysia dactylomela* Rang is known to sequester halogenated secondary metabolites from its diet. Due to their physical disabilities, this mechanism is extremely crucial for the organisms to escape or defend themselves from predators. The naturally occurring organohalogens in the marine environment are a major contributor to the diversity of halogenated metabolites within marine organisms. However, the proportion of halogenated metabolite in sea hare does not reflect the concentration of resulting metabolites from its diet source. Findings show that the concentration of metabolites in the organism is often much lower than its dietary algae and tends to reduce in between diets [28].

Brominated compounds are more prominent compared to chlorinated compounds in the ocean, while iodinated and fluorinated compounds are rare [29]. However, the ability of marine organisms to oxidize halogens enables the incorporation of these molecules into synthesized compounds [30]. Synthesis and bioaccumulation of secondary metabolites in marine organisms are necessary due to the ecological pressures within the marine ecosystem, including significant competition for space, deterrence of predation and a high level of symbiosis between different species. These compounds also contribute to growth, reproduction and defense. The amounts of these metabolites, though produced in low quantities, due their role in survival, rival those of primary metabolites [5]. The presence of halogens profoundly influences their biological activities, such as anti-bacterial, anti-fungal, antiviral, anti-inflammatory, anti-proliferative, anti-fouling, anti-feedant, cytotoxic, ichthyotoxic and insecticidal activities [31].

Secondary metabolites produced by marine organisms undergo a set of three phases involving functionalization and biotransformation prior to excretion via cell membrane. The first phase is where the metabolite is functionalized through the introduction of functional groups that reduce the lipophilicity of compounds via reactions: hydroxylation, hydrolysis, dehydrogenation, etc. Here, the cytochrome P450 monooxygenase (CYP) is a crucial enzyme that can detoxify molecules. The second phase involves biotransformation to enhance the hydrophilicity of molecules controlled by enzymes to promote glucuronidation, sulfonation, acetylation, methylation and conjugation with amino acids and glutathione. Eventually in phase three, molecules are transferred through cell membranes by transporter proteins for excretion [6].

However, recent development in experimental NMR techniques have addresed some challenges crucial to deciphering halogenated metabolites. As highlighted by Kutateladze and Reddy (2017), the utilization of ^35,37^ Cl isotope to affect the ^13^C chemical shift is an important tool for determining chlorine-bearing carbons. Computations of ^13^C chemical shifts have been instrumental to numerous structure revisions. Fast emerging parametric methods and the evaluation of nuclear spin-spin coupling constants, which was once thought to be difficult to compute, is allowing organic chemists to quickly solve and deduce misassigned structures successfully [32]. The thorough revisions of some major metabolites have been instrumental to critical revisions of secondary metabolites closely related to the sea hare. These revisions are taken into consideration throughout this review.

The reality is that plenty of research emphasis has been put on the discovery and characterization of new halogenated compounds and their applications. The ecological role of secondary metabolites has often been disregarded, although a few halogenated metabolites have shown important roles on the community structure in marine ecosystems. In some cases, chemical profiles have been used in the sorting of cryptic species. However, the chemotaxonomic value of natural halogenated compounds is often debated due to potential variations chemical composition according to geography and season. The following sections highlight the chemical constituents isolated from *Aplysia dactylomela* Rang.

## 5. Monoterpenes

**Figure 2:**
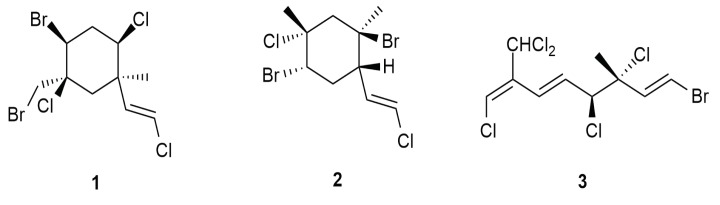
Chemical structures of monoterpenes from *Aplysia dactylomela* Rang.

The sea hare *Aplysia dactylomela* Rang has been reported to sequester bio-active monoterpenes, as detected in a specimen from Tenerife (Spain) [14]. The compounds 4-bromo-5-bromomethyl-2,5-dichloro-1-(E)-chloroethenyl-1-methylcyclohexane (**1**), 5-chloro-2-(E)-chlorovinyl-1,4-dibromo-1,5-dimthyl-cyclohexane (**2**) and (1E,5E,7E)-1-bromo-7-dichloromethyl-3,4,8-trichloro-octa-1,5,7-triene (**3**) were originally reported in the late 1970s from the red alga *Plocamium* sp. [33]. These compounds exhibited cytotoxicity towards cancer cell lines. Compound **1** displayed cytotoxicity towards HM 02 cell lines (gastric carcinoma), HEP G2 (liver carcinoma) and MCF 7 (breast carcinoma) at 17 µg/mL and lower. Compound **1** was also mildly cytotoxic towards the Lu1 cell line (human lung cancer) with an half maximal inhibitory concentration (IC_50_) of 12.9 μg/mL; KB (human oral epidermoid carcinoma) with an IC_50_ value of 13.3 µg/mL; and ZR-75-1 (hormone dependent human breast cancer) recording an IC_50_ value of 7.8 µg/mL. This compound also exhibited anti-algal potential by inhibiting the microalga *Chlorella fusca*. As an anti-tubercular agent, compound 1 recorded moderately potent activity with minimum inhibition concentration (MIC) values at 32 μg/mL and 34 μg/mL towards *Mycobacterium tuberculosis* and *Mycobacterium avium*. Compound **2** showed activity in the brine shrimp assay and screened for its nematicidal effects towards *Caenorhabditis elegans*. However, compound **2** did not display any form of nematicidal effects [14]. The chemical structures are shown in Figure 2.

## 6. Sesquiterpenes

### 6.1. Cuparane and Laurane Skeleton

**Figure 3:**
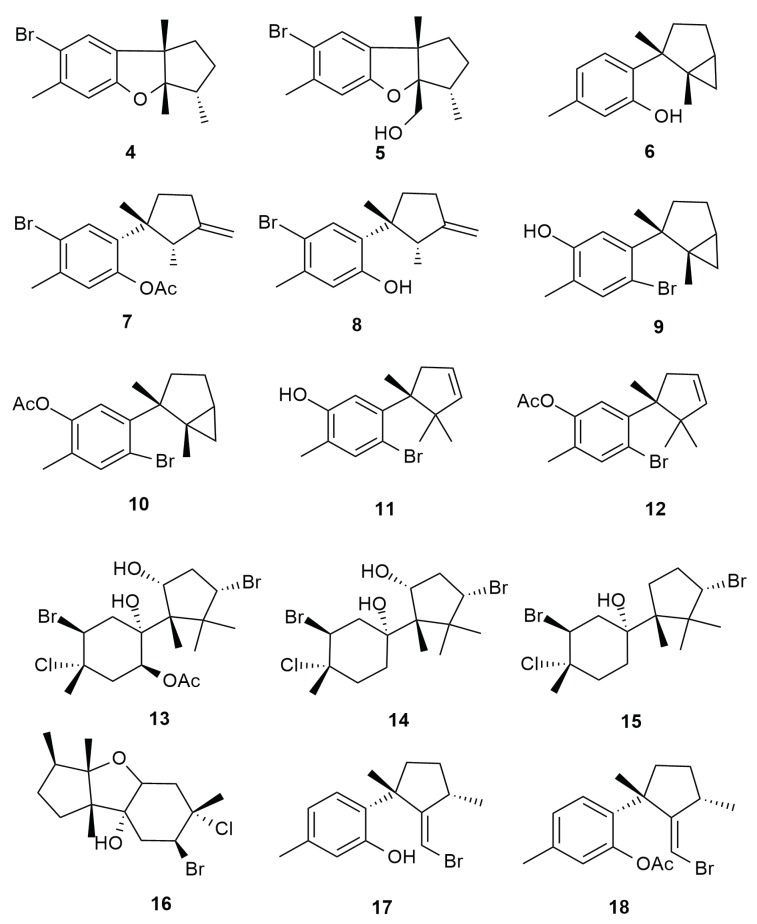
Cuparane and laurane type sesquiterpenes (**4**–**18**) from from *Aplysia dactylomela* Rang.

Cuparanes are formed by cyclization between carbons 6 and 11 of the bisabolane skeleton. In cuparane-type compounds, three methyls are present in the aliphatic portion at positions 1, 2 and 2, while in laurane-type compounds, the three methyls in the aliphatic portion are located at positions 1, 2 and 3 [34]. A study on the halogenated metabolites of *Aplysia dactylomela* Rang initiated with the isolation of brominated laurane type compounds, aplysin (**4**) and aplysinol (**5**) [35]; their source of origin remained unknown until Irie and co-workers (1966) isolated the laurane-type sesquiterpene laurinterol (**6**) from *Laurencia intermedia* [36]. Most sesquiterpenes are ecologically important as anti-epibiosis agents [19]. The isolation of laurinterol (**6** suggested that the compound aplysin (**4**) could be a product of transformation. Several years after, aplysin (**4**) and aplysinol (**5**) were isolated from *Laurencia okamurai*, indicating a possible source of the compounds in nature [37]. The occurrence of similar-in-nature metabolites became more common with the isolation of four halogenated non-aromatic cyclic laurane and cuparane type sesquiterpenes: algoane (**13**), 1-deacetoxyalgoane (**14**), 1-deacetoxy-8-deoxyalgoane (**15**) and ibhayinol (**16**) from the South African *Aplysia dactylomela* Rang [38]. The isolation of allolaurinterol acetate (**7**), an isomer of laurinterol (**6**) indicated the presence of cuparane type compounds in *Aplysia dactylomela* Rang. Apart from laurinterol (**6**), allolaurinterol (**8**) was also successfully isolated. The secondary metabolites allolaurinterol (**8**) and isolaurenisol (**17**), and their respective acetates (**7**, **18**) were also isolated from a New Zealand species [39]. The metabolite isolaurenisol (**17**) had been previously reported from the red alga *L. distichophylla* suggesting it to be a preferred diet by the investigated sea hare species [40]. The compounds allolaurinterol (**8**) and isolaurenisol (**17**) were reported to display moderate cytotoxicity towards P388 and BSC-1 cell lines but exhibited promising anti-bacterial inhibition towards *B. subtilis* with diameters 9 mm and 10 mm at 120 µg. These compounds were only moderately potent towards the fungus *Trichophyton mentagrophytes*. *Aplysia dactylomela* Rang from Kohama Island in Japan was also reported to yield the halogenated cuparanes cyclolaurenol (**9**) and cupalaurenol (**11**) together with their respective acetates (**10**) and (**12**) [41]. The laurane and cuparane type sesquiterpenes (**4**–**18**) from from *Aplysia dactylomela* Rang are shown in Figure 3.

### 6.2. Bisabolene Type Skeleton

In the effort to look for pharmacologically active metabolites from sea hare, the halogenated bisabolene, deodactol (**19**), an antineoplastic compound, was isolated from isopropyl extract of the sea hare digestive gland. Deodactol (**19**) is an isomer to the compounds caespitol (**20**) and isocaespitol (**21**) that was previously isolated from *Laurencia caespitosa* [42]. Similarities between these compounds are suggested to be linked to their biosynthetic pathways. However, the compounds caespitol (**20**) and 8-deoxy-isocaespitol (**22**) were eventually reported to be naturally isolated from *Aplysia dactylomela* Rang of the Bimini Islands [43]. Caespitol (**20**), displayed weak cytotoxic properties towards HeLa cell lines (cervical cancer) with IC_50_ values ranging from 10 to 100 µg/mL. It also exhibited potential activity in the brine shrimp assay and nematicidal effects towards *Caenorhabditis elegans*. Following these reports, another halogenated bisabolene iso-deodactol (**23**) was discovered from the species [44].

Continuous search for halogenated metabolites from specimen of Tenerife further led to the discovery of more bisabolene type compounds, puertitol-B-acetate (**24**), caespitenone (**25**) and 8-acetylcaespitol (**26**), which were proposed as products of algal derived compounds acetylation [14]. Along with these secondary metabolites, Wessels also reported the previously isolated caespitol (**20**), along with furocaespitane (**27**), caespitane (**28**) and laucapyranoid A (**29**). The reported compounds were originally reported from *Laurencia* spp., suggesting the possible dietary choice of the sea hare. Eventually, in 2006, three more bisabolene type derivatives, aplysiadiol (**30a**), deschlorobromo caespitol (**31**) and furocaespitanelactol (**32**), were isolated from a Canary Island species sea hare [45]. Surprisingly, there has been contrasting report on aplysiadiol (**30a**) by Ojika which characterized the metabolite as a halogenated diterpene (**30b**) from *Aplysia kurodai* with the molecular formula C_20_H_31_BrO_2_ [46].

**Figure 4:**
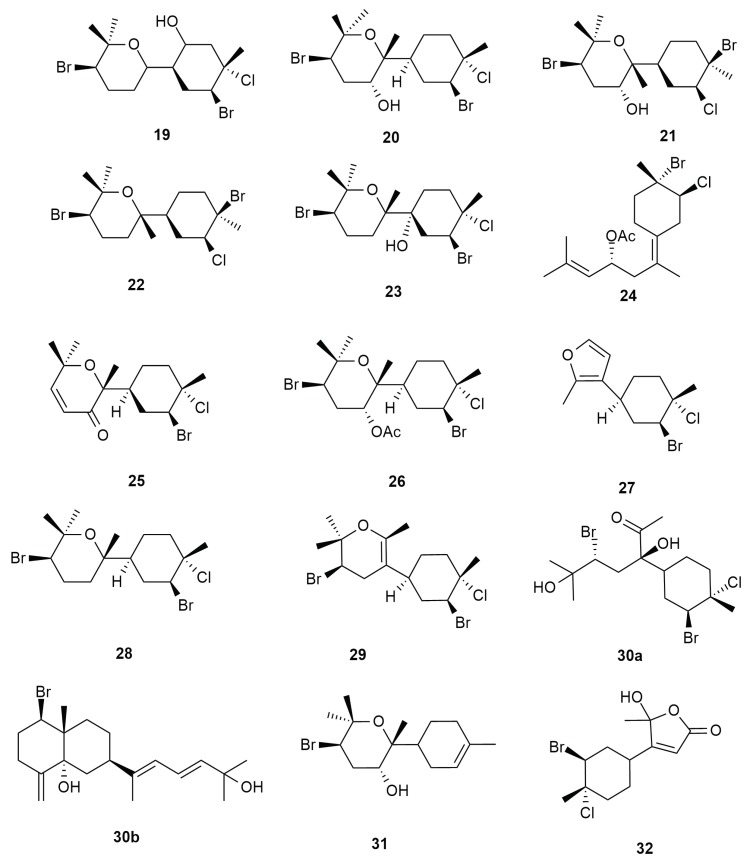
Bisabolene type metabolites that were isolated from the sea hare, *Aplysia dactylomela* Rang.

Figure 4 shows the bisabolene type metabolites that were isolated from the sea hare.

### 6.3. Syndrean Type Skeleton

**Figure 5:**
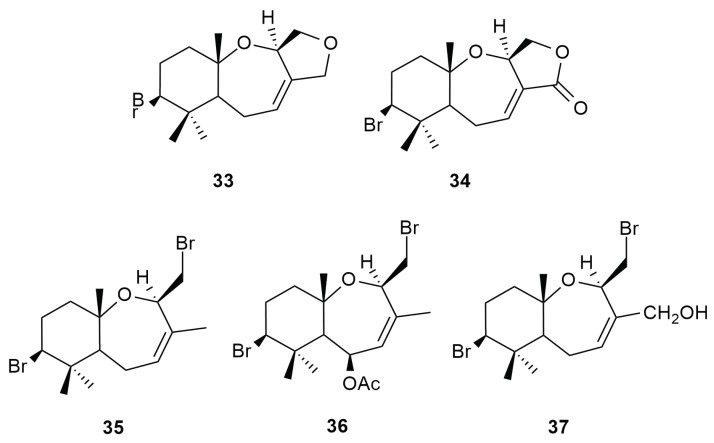
Syndrean type metabolites that were isolated from the sea hare, *Aplysia dactylomela* Rang.

In 2000, the isolation of syndrean type halogenated palisadin A (**33**), aplysistatin (**34**) and palisadin B (**35**) were reported from *Aplysia dactylomela* Rang [47] in an effort to study the dynamics of algal secondary metabolites in the sea hare [28]. Identical metabolites suspected to be chemotaxonomical markers to the red algae *Laurencia snackeyi* were also reported from *Aplysia dactylomela* Rang collected off the Bornean waters [10]. Aplysistatin (**34**) was found to be a potent antileukemic agent [48]. The mollusk from Borneo also yielded the compounds 5-acetoxypalisadin B (**36**) and 12-hydroxypalisadin B (**37**). The syndrean 5-acetoxypalisadin B (**36**) was previously reported as a product from the acetylation of 12-hydroxypalisadin B (**37**) [49] but was successfully isolated in its natural form in the sea hare of Sepanggar Island, Borneo. The isolation of these compounds confirmed that the sea hare of Borneo selectively feeds mainly on the red alga *L. snackeyi* [17]. This diet correlation helped a chemical ecologist from the Malaysian island to monitor local diversity of red algae in its waters. These syndrean-type compounds are unique to *L. snackeyi* (Weber-van Bosse), and were previously isolated by Paul and Fenical (1980) from *L. palisada*. Structures of these compounds are displayed in Figure 5.

### 6.4. Charmigrane Type Skeleton

The sea hare *Aplysia dactylomela* Rang is a rich source of charmigranes. Initial reports of charmigrane type metabolites were form several sources of the red algae *Laurencia*. Eventually, a wide range of charmigrane type halogenated metabolites began to be isolated from *Aplysia dactylomela* Rang. Literature in 2000 highlighted the isolation of halogenated charmigranes obtusol (**38**), cartilagineol (**39**), 9-acetylisoobtusol (**40**), elatol (**41**), 9-isoobtusol (**42**) and 9,15-dibromo-1,3(15)-chamigra-diene-11-ol (**43**) [14]. In the waters of Borneo, the compounds elatol (**41**) and 9-isoobtusol (**42**) are strongly associated with *L. majuscula* [17]. The compounds elatol (**41**); and 9-isoobtusol (**42**), its acetate dibromotrienol (**44**) and its isomer (**45**), were first reported fby *Aplysia dactylomela* Rang in the 80s [50].

Obtusol (**38**), elatol (**41**) and 9,15-dibromo-1,3(15)-chamigra-diene-11-ol (**43**) displayed cytotoxicity towards HM 02 cell lines (gastric carcinoma), HEP G2 (liver carcinoma) and MCF 7 (breast carcinoma) at concentrations lower than 17 µg/mL. Elatol (**41**) also showed cytotoxicity towards L6 muscle myoblast cells at 3.3 µg/mL and anti-trypanosomal activity towards *Trypanosoma cruzi* with IC_50_ of 0.92 µg/mL. It also exhibited cytotoxicity towards HeLa (IC_50_ 1.3 µM) and Vero cell lines (IC_50_ 25.0 µM) [51]. Cartilagineol (**39**) and elatol (**41**) were found to be cytotoxic towards A-549 (non-small lung cancer), HT-29 (human colon carcinoma), MEL-28 (melanoma) and P-388 cell lines (murine lymphoid neoplasm). Cartilagineol (**39**) recorded IC_50_ values 1.0 µg/mL, 0.25 µg/mL, 1.0 µg/mL and 5.0 µg/mL respectively, while elatol (**41**) recorded a consistent IC_50_ 0.1 µg/mL for each cell line, except P-388—1 µg/mL. Obtusol (**38**) and 9-isoobtusol (**42**) also displayed weak cytotoxic properties towards HeLa cell lines (cervical cancer) with IC_50_ values ranging from 10 to 100 µg/mL.

**Figure 6:**
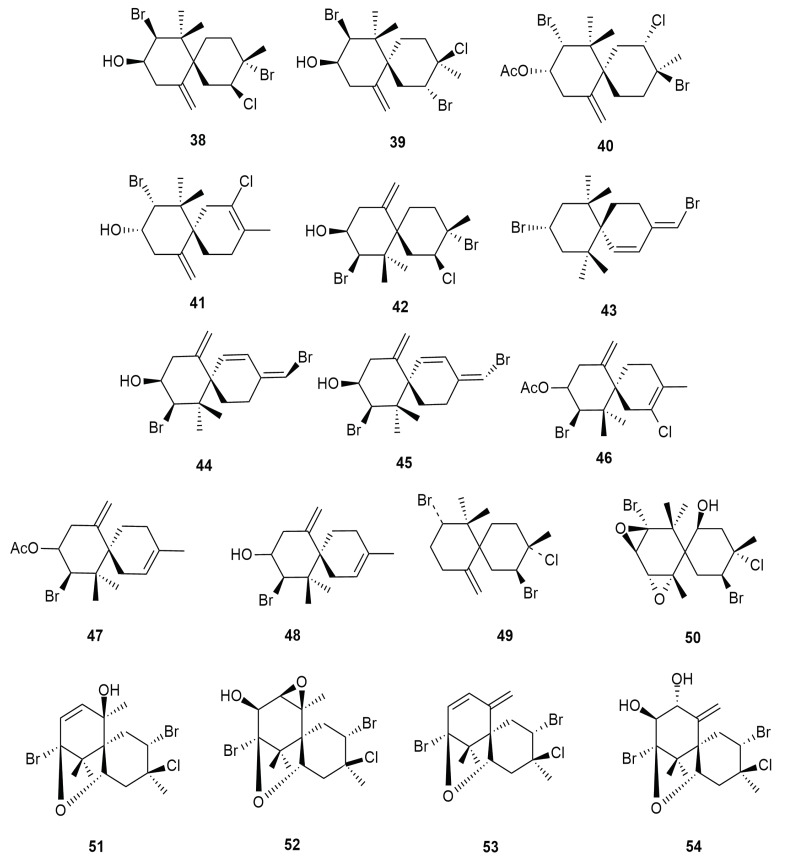
Charmigrane type metabolites from *Aplysia dactylomela* Rang.

Several derivatives of elatol (**41**) were discovered from the sea hare population of the Canary Island. The chamigranes acetylelatol (**46**) and acetyldeschloroelatol (**47**) were characterized. These compounds contained the halogens bromine and chlorine, and were reported to be cytotoxic [45]. Acetylelatol (**46**) exhibited cytotoxicity towards HeLa (IC_50_ 13.7 µM) and Vero cell lines (IC_50_ 44.6 µM) [51]. Dias et al. (2005) also characterized the compound deschloroelatol (**49**) which lacks chlorine. Investigation on the South African population of *Aplysia dactylomela* Rang yielded two additional charmigranes, nidificine (**49**) and prepacifenol epoxide (**50**) [38]. The presence of exo-methylene’s functionality in compound **49** is a contributing factor to the anti-viral attribute against the herpes simplex virus-1 (HSV-1) [52]. Sea hare populations from the Brazilian waters synthesized the halogenated charmigranes pacifenol (**51**), johnstonol (**52**), pacifidiene (**53**) and pacifenediol (**54**) [53], all of which are metabolites unique to *L. composita* Yamada. This data indicates the possible abundance of *L. composita* in the study location, thereby stressing the ecological importance of diet-derived studies. Figure 6 illustrates the chemical structures of halogenated charmigranes **38**–**54** from *Aplysia dactylomela* Rang.

Four charmigrane type sesquiterpene were isolated from the *Aplysia dactylomela* Rang specimen collected off Madagascar: (6S,10S)-10-bromo-3,11,11-trimethyl-7-methylidenespiro [5.5] undec-2-ene-4-one (**55**), (4S,6S,10S)-10-bromo-3,11,11-trimethyl-7-methylidenespiro [5.5] undec-2-ene-4-ol (**56**), (3R,4S,6S,10R)-10-bromo-3, 11, 11-trimethyl-7-methylidenespiro [5.5] undecane-3,4-diol (**57**) and (6S,7S,11R)-2-chloro-3,7,11-trimethyl-10-methylidenespiro [5.5] undec-2-ene-7-ol (**58**) were characterized [54]. Compound **55** is a derivative from the reductive debromination of the charmigrane dactylone (**59**). Compound **55** differs from dactylone (**59**) by configuration of the asymmetric center at C-10. NOE experiments determined difference in the ring A conformations in compounds **55** and dactylone (**59**). The difference between **55** and dactylone (**59**) can be observed also with the presence of chair configuration with an equatorial methyl group in ring A [55]. All four compounds isolated from the Madagascar sea hares were confirmed as the derivatives of dactylone (**59**) [56]. Compounds **56** and dactylone (**59**) were tested for cytotoxicity against HL-60 and THP-1 leukemia cells. Dactylone (**59**) did not exhibit toxicity, while **56** recorded IC_50_ values of 102 µmol/L and 152 µmol/L respectively. However, dactylone (**59**) caused apoptosis in both cell lines at an IC_50_ concentration of 80 µmol/L and 40 µmol/L respectively [54].

**Figure 7:**
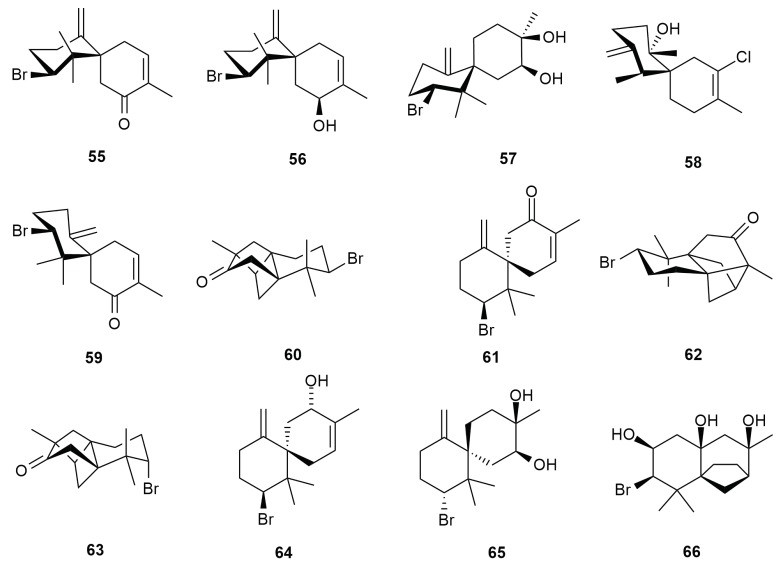
Additional charmigrane type metabolites **55**–**66** from *Aplysia dactylomela* Rang.

Dactylone (**59**) was first reported by Fedorov et al. (2000) along with its epimer 10-epi-dactylone (**61**) from the alcoholic extract of *Aplysia dactylomela* Rang [57]. Dactylone (**59**) was nontoxic against mouse epidermal JB6 P^+^ Cl41 cells and human lung cancer cells H460 at doses within the range of 150–200 µmol/L. It recorded a 50% inhibition at 45.4 and 92.4 µmol/L for both the cell lines respectively [58]. Aplydactone (**60**) was isolated from specimens of Nosy Hara, off the coast of Madagascar [59]. It possesses a highly strained, tetracyclic skeleton with three quaternary stereocenters, which are all embedded in the ladderane substructure [60]. It is assumed that dactylone (**85**) is converted to aplydactone (**60**) via photochemical addition due to the intense solar irradiation the sea hare is exposed to. Laboratory biosynthesis, however, revealed the need for a “biomimetic” light source at 350 nm for 24 h for the reproduction of aplydactone (**60**). Irradiation of 10-epi-dactylone (**61**) under the same condition produces 8-epi-isoaplydactone (**62**) rather than 8-epi-aplydactone (**63**), which was initially obtained via non-biomimetic synthesis [60]. Matsuura et al. (2017) also reported the metabolites 10-bromo-β-chamigren-2-ol (**64**) and 10-bromo-β-chamigren-2,3-ol (**65**). The stereochemistries of aplydactone (**60**) and 8-epi-isoaplydactone (**62**) are reported according to revisions by Kutateladze and Reddy (2017). One sea hare population from Canary Island yielded the compound dactylomelatriol (**66**), a naturally occurring omphalene-derived sesquiterpene. Dactylomelatriol (**66**) is the first example of a marine-derived omphalane sesquiterpene, which is believed to be derived from a chamigrene precursor. The only record of omphalene type compound in nature was reported as omphalic acid from the liverwort *Omphalanthus filiformis*. Like liverworts, seaweeds of the genus *Laurencia* possesses the ability to biosynthesize tricyclic sesquiterpene derivatives by internal cyclization of a chamigrene precursor. Formation of the omphalene skeleton is suggested to be related to the cyclication of a rhodolaurane intermediate. Theoretically, formation of rhodolaurane type metabolites in *Laurencia obtusa* makes it a possible source of origin. However, no concrete evidence is available [61]. Figure 7 illustrates the chemical structures of additional halogenated charmigranes **55**–**66** from *Aplysia dactylomela* Rang.

### 6.5. C-15 Acetogenin Type Skeleton

Acetogenins are molecules that are products of the polyketide pathway. In the marine environment, acetogenins are mostly halogenated and associated with marine algae and invertebrates, and exist from a common C-15 precursor derived from a C-16 fatty acid. They are proposed as possible chemotaxonomic markers for the red algae *Laurencia* [62]. In *Aplysia dactylomela* Rang, acetogenins are commonly found due to its diet correlation with red algae. It has synthesized the acetylenic dibromochloro ether, dactylyne (**67**), a chemical compound with a six-membered ring containing chlorine and bromine moieties [44]. Dactylyne (**67**) is also a compound known to prolong pentobarbital hypnosis in mice by inhibiting pentobarbital metabolism. Based on its halogenation, dactylyne is suggested to be of algal origin. In addition, isodactylyne (**68**), an isomer of dactylyne (**67**), was also isolated from *Aplysia dactylomela* in its natural form and characterized [63].

**Figure 8:**
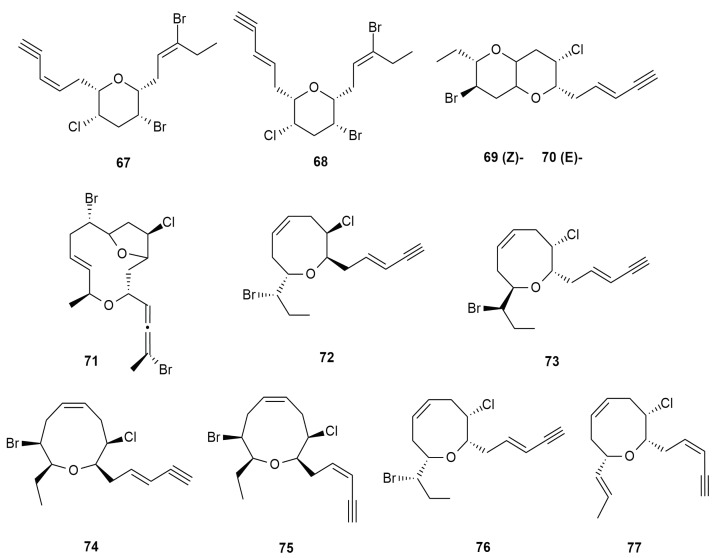
Diversity of C-15 Acetogenin type metabolites from *Aplysia dactylomela* Rang.

Gopichand (1981) isolated a pair of isomeric halogenated ethers, (E)-dactomelyne (**69**) and (Z)- dactomelyne (**70**), from the Caribbean sea hare [44]. Ten years later, a bromoallene type C-15 acetogenin, dactylallene (**71**), was isolated from the Atlantic *Aplysia dactylomela* Rang. Dactylallene (**71**) is a polyhalogenated bicyclic system with a 12-membered ether ring [64]. New C-15 acetogenins were also reported from the Hainan Island sea hare in the South China Sea, whereby (-)-3E,6R,7R-pinnatifidenyne (**72**); its enantiomer, (+)-3R,12R,13R-pinnatifidenyne (**73**), (+)-3E,6R,7R-obtusenyne (**74**); and (+)-3Z,6R,7R-obtusenyne (**75**) were characterized [65]. The enantiomer of a compound is determined when the optical rotation value is opposite to the other. Along with these additional two C-15 acetogenins, (+)-3-E-pinnatifidenyne (**76**) and (+)-laurenyne (**77**) were isolated. Acetogenins are more often associated to antibacterial activity towards different microorganisms, but their roles in both the algae and the sea hare are often questioned [62]. The chemical diversity of C-15 acetogenins isolated from *Aplysia dactylomela* Rang is shown in Figure 8.

### 6.6. Diterpene

**Figure 9:**
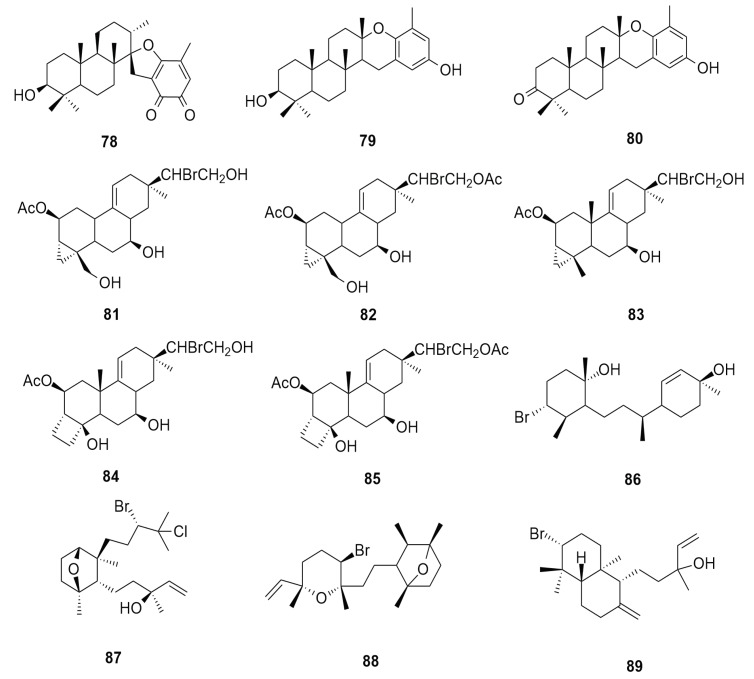
Diversity of halogenated diterpenes isolated from *Aplysia dactylomela* Rang.

Reports of diterpenes from *Aplysia dactylomela* Rang are scarce. Interestingly, there are random studies of the sea hare assimilating metabolites synthesized by brown algae. Few records claim that juvenile *Aplysia dactylomela* feed on algae besides *Laurencia*. Diterpenes isolated from this source are non-halogenated in nature. The diterpenes stypoldione (**78**), (+)-epitaondiol (**79**) and 3-ketopitaondiol (**80**) were previously only reported from the brown seaweed *Stypopodium zonale* [56]. Structures of these non-halogenated metabolites are displayed in Figure 9. Halogenated diterpenes from *Aplysia dactylomela* were the compounds with cyclopropane and cyclobutane rings. Four parguerene type acetates, acetoxytriol parguerol (**81**), parguerol-16-acetate (**82**), deoxyparguerol (**83**), isoparguerol (**84**) and isoparguerol-16-acetate (**85**), were isolated from the Puerto Rican *Aplysia dactylomela* Rang [66]. Sources of parguerenes have been previously reported from *Laurencia obtuse*, *L. filiformis*, *L. saitoi* and *L. nipponica* [67].

**Figure 10:**
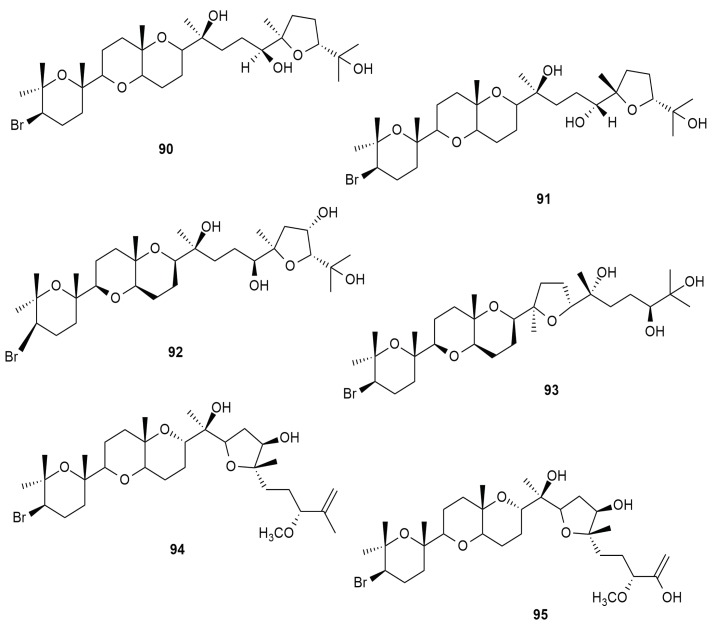
Halogenated tetracyclic triterpenes from *Aplysia dactylomela* Rang.

Further investigation led to the isolation of the diterpene 14-bromoobtus-1-ene-3,11-diol (**86**) from the *Aplysia dactylomela Rang* digestive tract. This compound is made up of an isoprene skeleton identical to cyclohexane and cyclohexene rings joined by a methyl-substituted trimethylene chain. The molecule possessed a bromine atom at the equatorial position in its cyclohexane ring. Cytotoxicity evaluation of **86** showed marginal 50% effective dose (ED_50_) inhibitory activity against the KB ( 4.5 pg/mL) and PS (10 pg/mL) cell lines [68]. The extraction of *Aplysia dactylomela* Rang individuals from Tenerife (Spain) resulted in the isolation of dactylomelol (**87**), dactylopyranoid (**88**) and isopinnatol B (**89**). The isolation of dactylomelol (**87**) has never been reported from an algal source, suggesting it to be possible product of natural biosynthesis. Dactylopyranoid (**88**) was isolated as a new structure that was biogenetically related to the compound laurencianol of *L. obtusa*, derived from a “head-to-tail” arrangement of four isoprene units. Isopinnatol B (**89**) is a compound with a labdane skeleton that can be obtained from geranylgeranyl pyrophosphate biosynthesis [14]. Figure 10 shows the diversity of diterpenes from *Aplysia dactylomela* Rang.

### 6.7. Triterpene

*Aplysia dactylomela* Rang was reported as a natural source of triterpenes. The tetracyclic triterpenes thyrsiferol (**90**), venustratriol (**91**), aplysiol A (**92**) and aplysiol B (**93**) were isolated from a South China Sea sea hare population [12]. Several individuals collected off Puerto Rico were found to sequester the bio-active compounds aplysqualenol A (**94**) and aplysqualenol B (**95**). Aplysqualenol A (**94**), was screened against several cancer cell lines and displayed IC_50_ values 0.4 and 0.3 mg/L against SNB-19 CNS (human brain glioblastoma mul) and T-47D (human mammary gland) cell lines respectively. Aplysqualenol A (**94**) was also evaluated against the herpes simplex virus type-1 (HSV-1) and HSV-2, varicella zoster virus (VZV), human cytomegalovirus (HCMV) and Epstein-Barr virus (EBV). The triterpene exhibited a 90% maximal response (EC_90_) towards HSV-1, HSV-2, HCMV and VZV at the concentration of 4 μg mL^−1^. It was toxic against the Epstein-Barr virus (EBV) at the concentration of 0.08 μg mL^−1^ (EC_90_). Aplysqualenol A (**94**) portrayed anti-tuberculosis properties towards *Mycobacterium tuberculosis* H37Rv at 6.25 μg mL^−1^. Both the tripterpenes aplysqualenols A (**94**) and B (**95**) displayed anti-plasmodial activity against *Plasmodium falciparum* with IC_50_ values 11 and 18 μg mL^−1^ respectively [48]. These bio-active triterpenes make up of a tetracyclic skeleton [69]. Figure 10 shows the chemical skeletons of the halogenated tetracyclic triterpenes.

## 7. Conclusions

Halogenated metabolites play an important ecological role in the sea hare *Aplysia dactylomela* Rang as part of its defense strategy for its survival in the marine environment. To the best of our knowledge, there has been no review on the diversity of halogenated metabolites from this sea hare species. This review presents the diversity of halogenated secondary metabolites that have been bio-accumulated and synthesized via modification by the sea hare *Aplysia dactylomela* Rang. The array of chemical structures with bio-active potentials and new metabolites discovered from this invertebrate suggest that these organisms are constantly adapting to their environment, as a means to ensure survival. New metabolites are constantly being discovered, leading to the development of pharmaceutical drugs. Regardless, a lot still can be done to understand role of marine natural halogenated compounds in the environment. Isolation and characterization of metabolites, evaluation of bio-activities, understanding their ecological relevance, application in taxonomic identification and monitoring biogeographical variation are only fractions of the research scope. Modern cutting edge edge technology and techniques promote a wider horizon to explore with a diverse range of organisms to investigate in the depth of the ocean.
